# Quantifying impacts of internships in an international agriculture degree program

**DOI:** 10.1371/journal.pone.0237437

**Published:** 2020-08-17

**Authors:** Shida Rastegari Henneberry, Riza Radmehr

**Affiliations:** 1 Ferguson College of Agricultural Sciences and Natural Resources, Oklahoma State University Stillwater, Stillwater, OK, United states of America; 2 Department of Agricultural economics, Ferdowsi University of Mashhad, Mashhad, Iran; University of Macau, MACAO

## Abstract

The completion of a meaningful, hands-on international experience is a critical and required component of the Master of International Agricultural degree Program (MIAP) at Oklahoma State University. Understanding the impacts of the international experience/internship is important in designing a curriculum that well-prepares students for their personal, social, academic, and future professional life. Hence, the objective of this study is to evaluate the MIAP students’ international experiences and to determine the factors that impact international experience outcomes. The benefits of international experiences are divided into five outcome areas that include personal, interpersonal, academic, employment, and civic impacts. The data is collected through an online survey of MIAP students. The variance based partial least squares structural equation modeling is used to develop three separate models with the goal of statistically measuring academic, employment, and civic impacts. The results of this study show that interns believe that international experience had enhanced each of their abilities. The findings of all three models show that the Humphreys travel grant has a statistically significant effect on interpersonal impacts. Additionally, the length of internship has a statistically significant association with personal impact in all three models, while it has a statistically positive indirect impact on academic, employment, and civic impact models, which indicates the full mediation effect. In addition, the number of hours worked weekly during the internship is found to have a significant positive relationship with employment impacts.

## Introduction

Internships can play a prominent role in preparing students for their future career. Some believe that the applied (hands-on) experience gained during an internship is an essential complement to the academic skills and knowledge and an experience that students cannot gain within a classroom [1, 2]. An internship can provide students with an experiential learning educational component as well as a chance to gain insight into career opportunities, leadership and management, and it allows them to substantiate their career interests in addition to improving their resume. Furthermore, the students that are immersed in an international setting are expected to gain greater maturity and self-confidence which can positively enhance the quality of the workforce [3–5]. Research has shown that industry representatives prefer to hire graduates who have meaningful previous professional experiences [6]. Lerner [7] emphasizes the fact that an internship has a positive effect on improving students’ skills and in helping them to have a successful career. Davies [8] notes that an internship experience provides an ideal opportunity towards preparing students for successful careers by integrating knowledge and applied skills. English and Koeppen [9] show that students who completed internships performed better in theoretical courses as compared to non-internship students. This is because internships are expected to provide the opportunity to improve the student’s knowledge-base and motivation to learn.

From an internship provider (organizations) perspective, internship programs can be useful by helping the host organizations establish and develop a connection with universities, using the students’ academic experience and knowledge to improve their organization’s performance, and at the same time gaining part-time employees [10, 11]. Turning to the other side of the argument, Lang [12], Chandra and Paperman [13], Beard [10] determine that internship programs may produce negative effects on interns. For example, Fox [14] found that unsuccessful experiences during an internship program could lead to a change in the student’s career path. Nevertheless, a negative internship experience could turn out to be a positive learning experience about one’s future career path in learning early on not to take that path. Hite and Bellizzi [15] indicate that there is a close connection between the lack of understanding that host organizations have of students’ expectations from their internships and the disappointing results that internship providers experience from the students’ work during their internships. Wood [16] finds that the better the organizations’ understanding of what students expect to gain from their internship and consequently adapting their internship descriptions and duties, the more positive the outcome of the internships will be. Some host organizations do not have a proper understanding of the learning needs of the students[17] and therefore students are often dissatisfied, and they complain about the quality of their internship because the internship programs are not well organized and structured [18, 19].

The Master of International Agriculture Program (MIAP) at Oklahoma State University (OSU) was created in 2008 as a multidisciplinary degree program. One of the advantages of MIAP is that it reduces the learning gap between theory and reality by enhancing the applied part of the students’ education through the international experience component of the program. MIAP has two distinct degree programs: Master of Science (MS) and the Master of Agriculture (MAG), both in International Agriculture. MIAP allows students the opportunity to choose a focus area that fits their personal, professional, and academic goals. Degree plans of MIAP students may include courses from various subject areas which can be offered by different departments, and even from different colleges. Some example focus areas are: rural development, agricultural entrepreneurship, community engagement and sustainability, and production agriculture and food security. Upon approval of their academic advisory committee, students may also custom design their own focus area. A MIAP degree can be completed either on campus, completely online, or through a combination of both. MIAP, despite offering only a master’s degree, has become one of the largest graduate degree programs (master and Ph.D. enrollment combined) in agriculture at OSU. While the study of international agriculture is at the core of its discipline, the MIAP program also seeks to provide its students an opportunity to explore other areas of growth, including enhanced leadership and organizational skills, professional communication, and project management. Graduate degree candidates are expected to complete a four-week or longer international experience that allows them to apply principles learned in the classroom in a given focus area (e.g., agritourism, education, international markets and trade, international development, or sustainability). If the students’ international experience is of eight weeks or longer, they qualify to apply for a generous travel grant (Humphreys—HU) for their international experiences. Because the internship is such an integral part of the education of MIAP students, evaluation of the effectiveness of the hands-on experience is a necessary part of the learning outcomes assessment of MIAP. The evaluation of the effectiveness of international experiences (internships) could provide useful information that is necessary for curriculum design and planning. Hence, the objective of this study is to evaluate the effectiveness of MIAP students’ international experiences and to identify the factors that determine the short- and long-term impacts associated with international experiences. The results of this research is expected to contribute to the growing body of research on the evaluation of experiential learning. In addition, this study is expected to provide information that could be used by internship organizers at academic institutions as well as internship providers in host countries in designing internships with positive impacts on students as well the host communities.

The rest of the paper is organized as follows: in the second section, a literature review is provided. In the third section, research model and hypotheses are presented. Then, the research methodology is discussed, followed by the analysis of data and empirical results. The final section offers a discussion of results and conclusions from findings.

## Literature review

**Fig 1:**
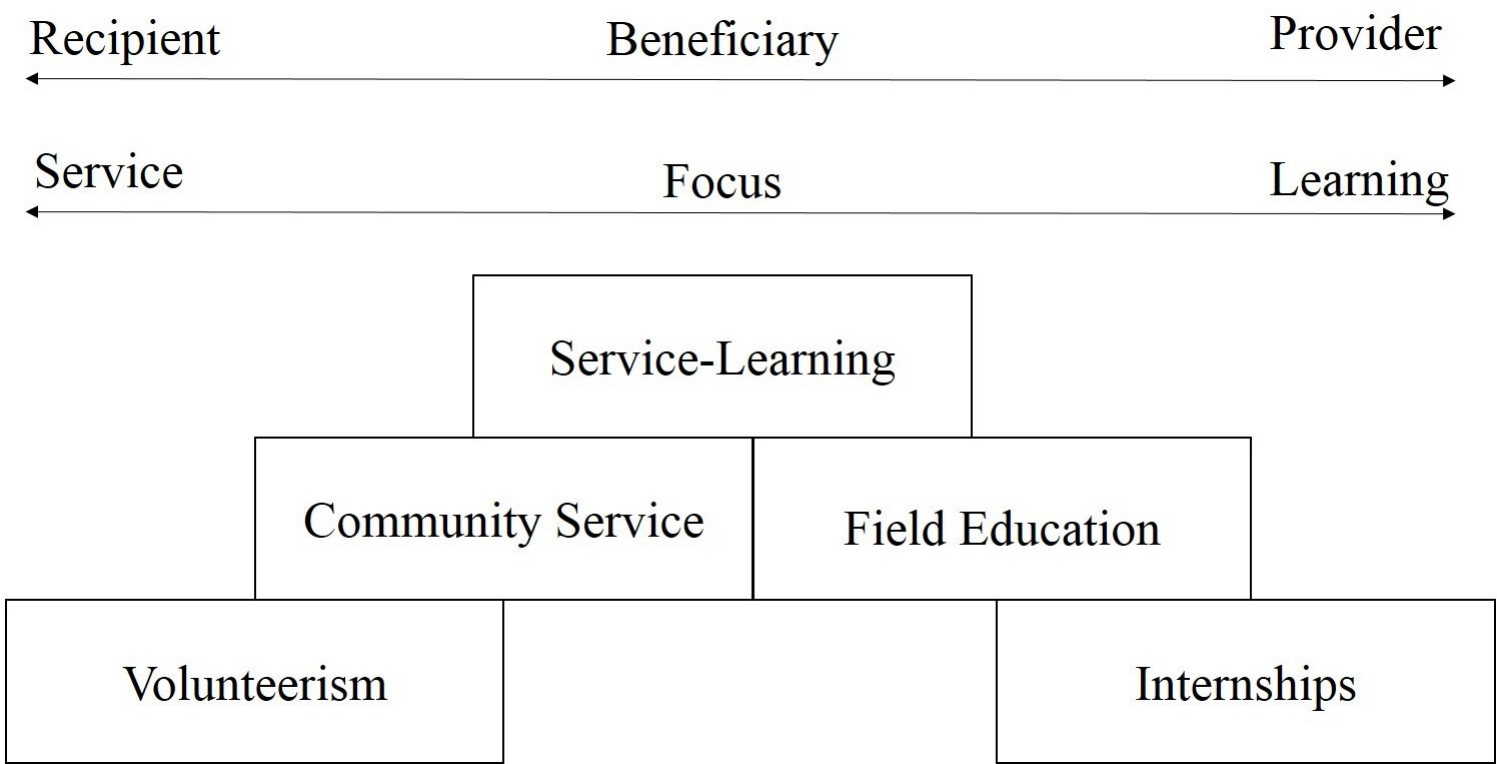
Distinctions among service programs [20].

First, in order to classify the MIAP’s international experience, it is important to determine the distinction among different types of community-based activity and experiential learning. For this purpose, we used Furco’s continuum [20] (see Fig 1). Fig 1 shows that service programs can be classified into five different experiential learning practices (volunteerism, community service, service-learning, field education, internships) by their intended beneficiary and goal of learning. As can be seen, in a volunteer and community service program, the emphasis on activities that provide a service and intended beneficiary is the recipient of service. On the other hand, field education (this service is performed as part of students' academic program to improve students' insights into their field of study) and internship (this program is aimed to provide opportunities for students to prepare themselves for their future career by gaining experience in a career field. Internships, which may be paid or unpaid [20], are focused on learning and the primary intended beneficiaries are the students. The service-learning is located in the center of Furco’s continuum which shows there is a balance between beneficiary and student learning. Jacoby [21] defines service-learning as “a form of experiential education in which students engage in activities that address human and community needs, together with structured opportunities for reflection designed to achieve desired learning outcomes" (P.2). According to the above and previous, the MIAP’s international experience can be classified into internship and service learning. Although the MIAP international experiences might fall into the *internship* category, the majority of them have characteristics of *service learning*. An important aspect of the MIAP international agricultural experience is that while it is an internship program and thus a benefit to student, many of the internships also feature service-learning characteristics and confer benefits to the host communities. Impact statements from students indicate that these opportunities have provided life-changing experiences for the student participants and also indicate that the students believe they have had an impact on the communities. For example, MIAP students who go to Uganda prepare in advance by learning the agricultural course modules that they will be teaching or by learning about agricultural production in Uganda. Through their international experience in Uganda, students serve the women and youth in the agricultural communities by teaching agricultural production during their two months of stay. Part of the student teaching is in the field and part in a classroom setting. These students advance the practical knowledge of the youth and women in the villages that they are placed in. MIAP students with Agribusiness background have been put into work identifying the most profitable marketing channels for the excess supply of crops and also do book keeping and accounting. Another good example of a MIAP service learning component is the projects that MIAP students are assigned to in Peru. Students work with the farmers belonging to the Farmers’ Association of Piura. Examples of projects that students are involved in include soil testing as wells as the identification and treatment of crop diseases. Students with agricultural communication undergraduate background have done marketing flyers and have designed promotion activities for the organizations that their international experience involves with. For example, one student helped with the advertising and design of e-brochures for the Australian Egg Corporation. Two students completed an internship designing marketing material for a vinery in Italy.

### International experience

The globalization and the need for intercultural communication skills have triggered changes in the design of new and existing educational programs intended for training professionals that are prepared to work in a global environment [22, 23]. In addition, today more than ever, students are seeking educational programs that teach them the skills to work in an international environment [24]. In response, many academic degree programs have incorporated international experiences as part of their degree requirement in order to provide an opportunity for students to experience work in the real-world, different cultures, and system [25]. The number of US students going abroad for international experience has been on the rise. IIE (Institute of International Education) reports that the number of U.S. students participating in international experiences has increased by 185% in 2012 compared to 2000 [26].

These programs allow students to develop personal and professional skills through a process of observation, reflection, and experimentation [27]. Zhang [28] points out that international experience is considered to be a type of experiential learning which not only provides a great opportunity for learning and gaining experience in a scientific field, but it is also one of the best ways to get acquainted with the culture, customs, indigenous knowledge, and languages of other countries. Cutler and Borrego [29] examine the benefits of an international internship for doctoral degree in a IGERT (Integrative Graduate Education and Research Traineeship) program at Virginia Tech. Their findings show that international experience has personal, academic, and employment benefits. For example, interns stated that the international experience improved their ability to live independently. Moreover, Cutler and Borrego [29] point out that interns tend to accept a permanent position or a post-doctoral training outside of the U.S. Divine, Miller [30] aim to identify the student characteristics that were improved during their international experience. Their findings show that engaging in an international experience did not affect students’ reliability or communication skills, but did significantly improve students’ interpersonal skills, ability to handle stress, and attitude.

### Experiential learning

As mentioned earlier, the MIAP international internships fulfill the experiential learning requirement of the degree program. Many studies have shown that experiential learning is an effective means of helping student to learn. Therefore, it is important to provide the various definitions of this term that have been used in the existing literature. Experiential learning has been defined in many ways in the literature. The first definition of experiential learning could be attributed to Rogers [31], who defined this term as “It has a quality of personal involvement, the whole-person in both his feeling and cognitive aspects being in the learning event” (p. 5). Experiential learning was defined by Hoover and Whitehead [32] as “when a personally responsible participant cognitively, affectively, and behaviorally processes knowledge, skills, and/or attitudes in a learning situation characterized by a high level of active involvement” (p. 25). Moreover, in other studies, the term experiential learning has simply been defined as an opportunity of outside-the-classroom learning, or in other words, “learning by doing” [33].

Regarding its impact on student learning, previous studies have emphasized the importance of experiential learning in improving the quality of agricultural education system [33–36]. For example, agricultural educators recognized several advantages of experiential learning, including the increasing importance of theoretical topics among students, active interaction, and improved problem-solving ability [37]. Among different types of experiential learning, the internship is often a critical component of management education [38].

In this study, impacts and benefits of experiential learning are divided into five outcome areas. These areas were extracted from several sources: [39–43]. Five categories of learning impacts were selected for this study: personal (PI), interpersonal (social, SI), academic (AI), employment (EM), and civic impacts (CI). The personal impacts are related to the individual’s motives, thoughts, and feeling about themselves as the results of experiential learning. Several scholars have argued that experiential learning can contribute to a change in personal outcomes [42, 44]. A study by Simons and Cleary [40], shows service learning as experiential learning provides practical experience to improve the students' personal development, such as self-knowledge. Regarding social impacts, the findings of several studies show that experiential learning plays a critical role in promoting awareness about cultural diversity, society’s concerns and problems, and reducing many of the failed and unacceptable cultural beliefs, such as ethnocentrism [25, 42, 45]. For instance, Prentice and Robinson [46] discovered that service learning provides a proper opportunity for the students to identify their own faulty beliefs about people and the society in which they live. These experiences allow students to acquire accurate information about other cultures, to develop effective communications skills, and to have empathy and compassion for other individuals.

Additionally, results of previous studies have indicated that experiential learning is associated with positive academic outcomes, such as final grades and degree classes [47–51]. Markus, Howard [52] pointed out that students who participate in experiential learning have a higher level of classroom learning and a higher course grade as compared with other students. In other words, the applied learning program can compensate for most of the weaknesses of the classroom instruction. Students’ participation in experiential learning provides a more comprehensive insight into scientific theories in the classroom.

Past studies have shown that employment impacts are an important criteria when measuring the effectiveness of internships [53–55]. For example, work experience gained during an internship is a valuable attribute in landing a good job and in career success [53, 56, 57]. Experiential learning, such as an internship, provides a proper opportunity for students to implement scientific theories they had learned during their classroom education.

Civic impacts deal with participants’ civic awareness, levels of civic engagement, and social responsibility. Knapp, Fisher [41] show that students who participated in an experiential learning program tend to become more effective citizens in their communities. Lee, Olszewski-Kubilius [58] point out that the applied learning program has a positive and significant impact on having a stronger connection and commitment to the community.

### Factors that determine the outcome of students' internships

Past studies have shown that intern and internship characteristics such as age, gender, marital status, employment status, the number of hours involved during an internship, and financial support, are considered important in determining the outcome of an internship. For instance, Ju, Emenheiser [59] indicate that interns’ level of satisfaction with their internships were influenced by factors such as age and gender as younger people and females report less satisfaction with their internships. Jackel [30] shows that gender (males) has a positive and significant correlation with employment and civic impacts of the internship. Besides, Jackel [30] shows a positive correlation between marital status (married) and civic impacts. On the other hand, Hergert [60] and Luecking and Fabian [61] found that gender does not correlate with the internship outcomes. However, the above-mentioned studies [30, 48] do not infer to causality, but rather a simple correlation between intern characteristics and the internship impacts on them.

The length of the internship experience is another component that has been shown to affect the internship outcomes. Petrillose and Montgomery [62] show that increasing the length of the internship program plays a critical role in improving the interns’ skills and abilities. According to the findings of this study, about 71% of students believe that the ideal length of an internship program is 1200 hours or six months. That is, a longer internship program provides a better opportunity to the participants for gaining full benefits from their internships. Phoebe [63] shows that the length of the internship has a positive and statistically non-significant impact on the effectiveness of the internship.

Moreover, research has shown that financial support provided to the students who attend an internship program could be considered as a key component influencing the effectiveness of the internship. In fact, interns who receive a higher level of financial support are shown to be more satisfied with the internship program [64]. Financial support allows students to better focus on learning from their internships. Litalien and Guay [65] show that research grants and scholarships have a significant effect on student perseverance in internship programs. Also, Weidman and Stein [66] found that financial support enhances students' commitment and responsibility to a research team, hence has a positive social impact on students. Furthermore, Attfield and Couture [67] indicate that 91% of students who attend an internship program are strongly in need of complementary financial support during their internship. Also, 48% of the interns who did not have financial support were depended on the support of parents, spouses, and relatives, and 38% of the interns had to find other employment to cover their costs. The findings of their research indicates that students who received financial support had about four times less dependence on outside financial support than other students. Hence, it is expected that the financial support of interns will have a significant effect on all five internship impacts (personal, interpersonal, employment, academic, and civic).

## Research model and hypothesis

The intern and internship characteristics included in this study are employment status during the internship (PEM), employment status in the current situation (CEM), the number of hours worked weekly at internship (HO), length of internship (WE), Humphreys travel grants (HU) (HU is considered as a proxy for financial support), access to supervisor during internship (SUP), and preparation and or training received before placement (TRA). In the first step and in order to design the research models, we selected those characteristics that were shown from previous studies (mentioned above) to have a significant correlation with the internship impacts. For this purpose, we employed the Pearson correlation coefficient to test the strength of possible associations between intern/internship characteristics and internship impacts. According to the results of the Pearson correlation coefficient (see S1 Appendix), length of the internship (WE) and working hours (HO) is shown to be positively associated with personal, interpersonal, academic, employment, and civic impacts. There is also a positive and significant correlation between Humphreys travel grants (a significant source of funding at OSU for international experiences), interpersonal and civic impacts. In addition, access to supervisor during internship (SU) is positively and significantly correlated with personal and civic impacts. There is a negative and significant correlation between employment status during the internship (PEM) and civic impact.

**Fig 2:**
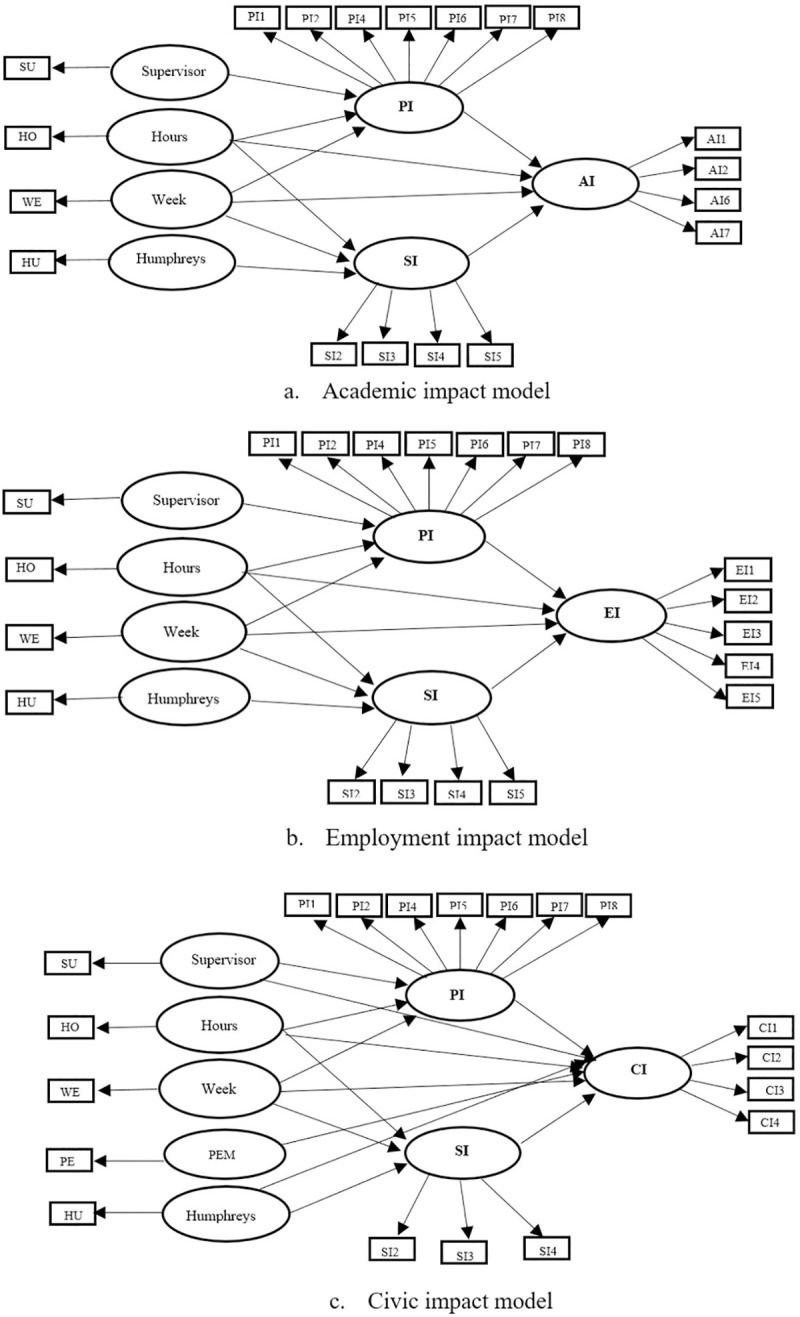
Research models.

Stelljes [68] and Jackel [43] found that the impacts of experiential learning are first reflected in personal and social empowerment, and then the two forms of empowerment have a mediation effect on learning outcomes (employment, academic, and civic benefits). Hence, it is expected that personal and social impacts will have a mediation effect on the relationship between student intern/internship characteristics and academic, employment, and civic outcomes. Finally, according to the findings of previous studies, the research model is constructed (see Fig 2). Consistent with the literature review and our research model, the following three hypotheses is proposed for this study (see Table 1).

**Table 1:** Proposed hypotheses in this study.

**Academic impact model’s hypotheses**	
H_1_	The personal and interpersonal (social) impacts have a mediation effect on the relationship between student intern/internship characteristics and academic outcomes.
**Employment impact model’s hypotheses**	
H_2_	The personal and interpersonal (social) impacts have a mediation effect on the relationship between student intern/internship characteristics and employment outcomes.
**Civic impact model’s hypotheses**	
H_3_	The personal and interpersonal (social) impacts have a mediation effect on the relationship between student intern/internship characteristics and civic outcomes.

### Research methodology

Federal regulations and Oklahoma State University policy require review and approval of all research studies that involve human subjects before investigators can begin their research (Application Number: AG-18-47, Date: August 14, 2019). The Office of University Research Compliance and the Institutional Review Board at Oklahoma State University conducted the aforementioned review to protect the rights and welfare of human subjects involved in biomedical and behavioral research. In compliance with this policy, this study received proper surveillance and was granted permission to be executed.

### Instruments

The data for this study is collected through an online survey of MIAP students and alums. The survey questions are divided into three distinct parts. Questions addressing student interns’ gender, the focus of the internship, marital status, and job status, constituted the first part of the survey. The second section of the survey sought to gather host organizations’ information. The third section of the questionnaire, designed to gather information on the perceived impact of international experiences on student participant, is divided into five separate parts: personal, interpersonal, academic, employment, and civic impacts. These five parts are adapted from the questionnaires created by Jackel [43] and Baird [69]. This section is comprised of 32 items, out of which eight items belong to personal impact (PI) i.e. (1) I feel satisfaction in performing valuable tasks. (2) I have acquired skills that help me to make a difference. (3) I am open to new experiences. (4) I have gained the capacity to be more productive. (5) I am able to identify my personal strengths. (6) I am able to identify my personal weaknesses. (7) I feel a personal achievement. (8)I have acquired the ability to endure a hard working conditions. Five items belonged to interpersonal impact (SI) i.e. (1) I express worry regarding the welfare of community. (2) I can be understanding and appreciative of people with diverse backgrounds. (3) I have the ability to communicate effectively. (4) I have improved my capacity to become a leader. (5) I feel more connected to my community. Seven items belonged to academic impact (AI) i.e. (1) I have gained experience from the tasks I performed during my internship. (2) I have developed my critical thinking abilities. (3) My grade point average was improved. (4) I desired to continue my education for another master or a Ph.D. degree. (5) I have the ability to work and learn independently. (6) I believe that international experiences improved my classroom learning. (7) I have acquired abilities to relate scientific topics to the real world.

Further, six items belonged to employment impact (EI) i.e. (1) I developed specialized technical skills for a specific job function(s). (2) I believe that my hands-on knowledge was improved. (3) My future career prospects were enhanced. (4) I received an opportunity to explore a specific career. (5) I have expanded realistic ideas about the workload. (6) I narrowed my future possible career choices.

Six items belonged to civic impact (CI) i.e. (1) The internship assisted me in being a better citizen. (2) The internship provides a deeper understanding of problems and concerns of the community. (3) I have acquired the ability to make a difference in my community. (4) I have acquired the capability to contribute to society. (5) I dedicate more time to volunteer work. (6) I stay current with the local political/social/economic news of the country of my internship.

In this section, we used a modified Likert scale, ranging from A great deal = 5, Quite a bit = 4, Moderately = 3, Slightly = 2, to Not at all = 1. The mean of these points is 3. A mean of 3 or above shows agreement with the proposed item statement; while 3 or below indicates disagreement.

The factor analysis is performed for each internship’s impacts separately and the items that had loading below the threshold of 0.7 were omitted. The omitted items included: one item from personal, one from interpersonal for academic and employment models and two from interpersonal for civic model. In addition, three were omitted from academic impact (AI), one from employment impact (EI), and two from civic impact (CI).

### Sampling

The target population for this study is composed of MIAP alums and current students (as of fall semester 2019) who had completed an international experience/internship abroad between 2008 and 2019. It is worth noting that for the purpose of this study an international experience is defined as lasting for a period of a minimum of four weeks and its location being outside the student’s home country. Hence, for U.S. citizens and U.S. resident student participants, host organizations were located outside of the U.S, while for international participants they were inside the U.S. (see Fig 5). To collect data, an online survey instrument was employed. This survey was designed through Google Forms and it was distributed using an embedded link through email in two rounds, first in October 2018 and then again in October 2019 in order to increase the sample size, as the number of respondents was not sufficient during our first round of survey in 2018. The online survey was activated during the first round from October 8, 2018, to October 30, 2018, and again in its second round from October 28, 2019 to November 11, 2019. It is worth to note that in order to increase the sample size, we made a strong effort and invested time in compiling current and active email addresses for those alums which their university email addresses had become inactive because of their lack of use. We then re-sent the survey (second round) to the updated email addresses and to current student in 2019. The second round of survey also included 2019 enrolled students (having sent the first round of survey in 2018, the list serve did not include 2019 enrolled students). We also sent multiple reminders, encouraging response from those who had not responded before.

Respondents were able to follow the link of the survey and display it in the web browser. The completed surveys were submitted electronically, the results were transmitted to Google Forms and finally were exported to Microsoft Excel 2013. The survey was sent to 129 MIAP alums and students. Each of the 129 students/alums had completed a significant international experiences between 2008 and 2019 and while a MIAP student. From the 129 surveyed alums/current students, a total of 85 responses were received, resulting in a 66% response rate. We then applied the 85 observations (sample size) to each of the three models (academic, employment, and civic impact models). It is important to note that the estimation process for each model are completely independent of the other two models.

### Analytical methods

A Structural Equation Modeling (SEM) is used in this study to analyze the impact of international experiences on MIAP students and alums. A brief explanation of SEM is given in this section. This method enables modeling, simultaneous estimation, and testing of complex theories regarding human behavior [70]. The structural models contain observed variables and latent variables. For example, in the models in this study (see Fig 1), PI1 is one of the observation variables (rectangles), and PI is one of the latent variables (ovals). The SEM is remarkably different from the traditional statistical approaches such as regression methods. The regression analysis has been used to model the individual observations where estimated coefficients are obtained by minimizing the sum of the squared residuals. Conversely, SEM models the covariance matrix of the observed variables [71]. In terms of the number of equations, SEM is usually defined by two or more equations in the model which differs from the common single equation regression that has a dependent variable. In addition, in the SEM, dependent variables in one equation may be considered as independent variables in another equation. Hence, the variables in this approach are defined as either endogenous or exogenous variables [72].

The partial least squares structural equation modeling (PLS-SEM) is one main method for estimating the SEM [70]. The PLS-SEM is employed to analyze the causal relationships between variables. This model is a multivariate technique that maximizes the explained variance of dependent variables. PLS-SEM is recommended for small sample size data [73, 74]. This approach is in contrast to covariance-based structural equation modeling (SEM) that having a large sample size is one of the preconditions for using this model [75]. Also, PLS-SEM does not need the assumption of multivariate normality [76] and results in this approach remain robust despite the existence of missing and outliers observations [77].

One of the most important reasons for employing PLS-SEM are related to the small sample size [78]. It is concluded in several published studies that using PLS-SEM is able to efficiently solve the problems of small sample size [74, 75, 79, 80]. For instance, Andrei, Zait [74] used PLS-SEM to analyze the data of two groups of participants with sample size of 42 in each of the two groups. In addition, based on the findings of Wang, Wallace [75] the results of PLS-SEM with sample size 32 for social- comparison cooperation were reliable and acceptable. Hence, due to the ability of PLS-SEM to analyze data with small sample size and other advantages which are mentioned above, PLS-SEM was employed in this study. We used Stata [81] software as the analysis tool in this study to test research hypotheses. In this study, in order to evaluate the path significance, we applied a bootstrap resampling procedure [82] with 1000 subsamples.

The K-Nearest Neighbors (KNN) model is a practicable imputation method for missing Likert scale values in small data sets [83], and it is used for cases where the number of missing values is less than 5% of the total data used [84]. Since the number of missing data in this study is 2.3% of the total data, the KNN model is employed in our study to deal with missing values in order to enable us to include the responses from all the individuals who had responded. Employing this method led to an increase in the sample size used in the analysis. The KNN approach is a powerful tool that has received considerable attention. It has been successfully applied in several empirical studies [84–87].

## Results

In this section, the results are presented in three broad categories: descriptive statistics, the intern’s perceived impacts, and evaluation of PLS-SEM. Detailed results are reported below.

### Descriptive statistics

#### Personal information

The survey sample population used in the analysis of this study follows the MIAP population in terms of gender and US citizenship/residency. Survey results show that 62% of respondents are female, while the rest are male (38%). This is compares with MIAP alum statistics where female is 60% of total MIAP alum population of 129.Therefore, the sample could be considered as a representative of the MIAP population.

Regarding employment status, the survey results show that a large percentage (85%) of respondents were employed full-time at the time of survey collection, while the rest were unemployed (15%). Furthermore, about 38% of respondents were employed full-time during the period of their international experience.

**Fig 3:**
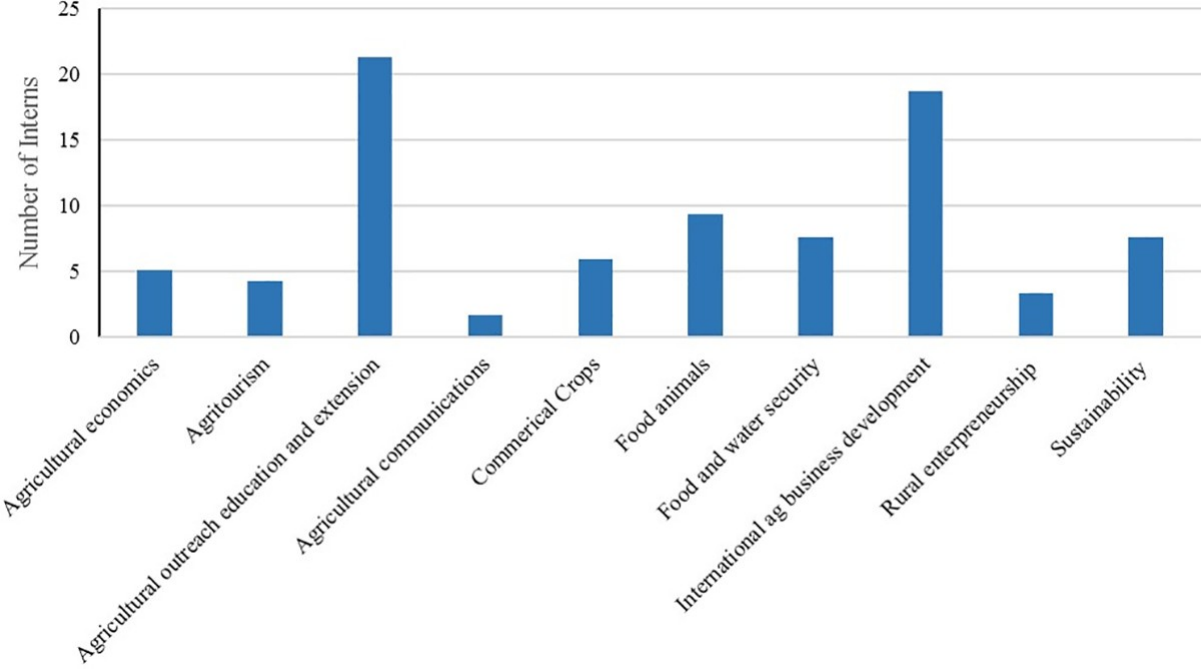
Bar chart showing the focus area of international experience.

With regard to the focus of international experience, survey results show that almost half of respondents completed their international experience in two fields: “Agricultural Outreach Education and Extension” and “International Agricultural Business Development” (See Fig 3).

### Internship program information

**Fig 4:**
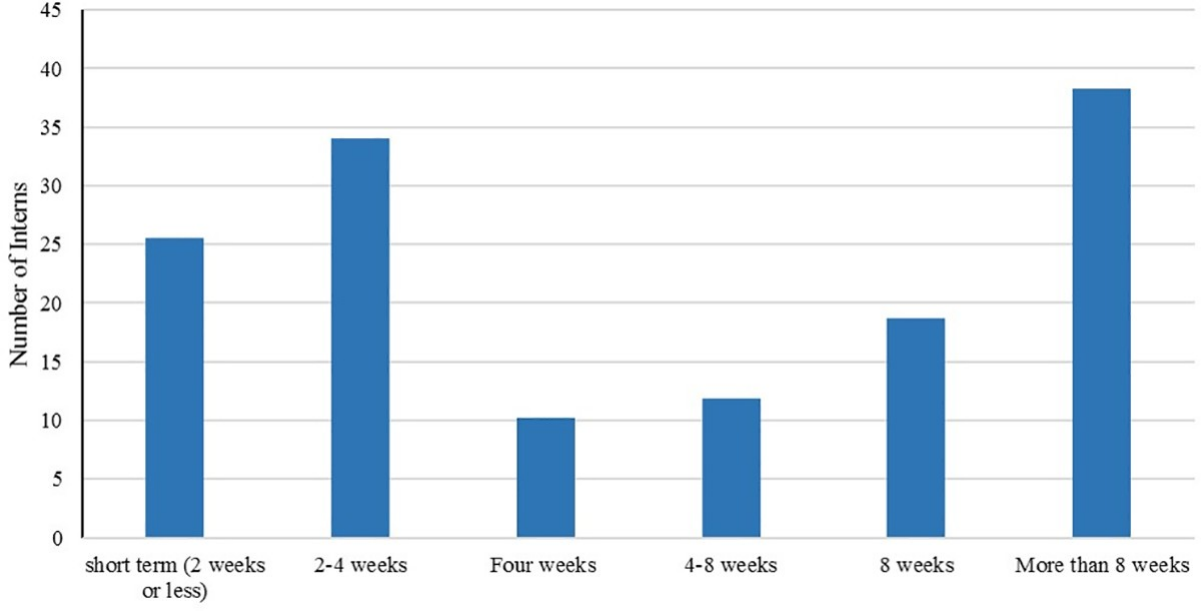
Bar chart showing the length of the international experience in weeks.

As evidenced in Fig 4, almost two-thirds (67%) of respondents reported that the length of their international experiences were “8 weeks” and “More than 8 weeks” while about 7% of respondents completed their international experiences in less than the expected length (4 weeks).

**Fig 5:**
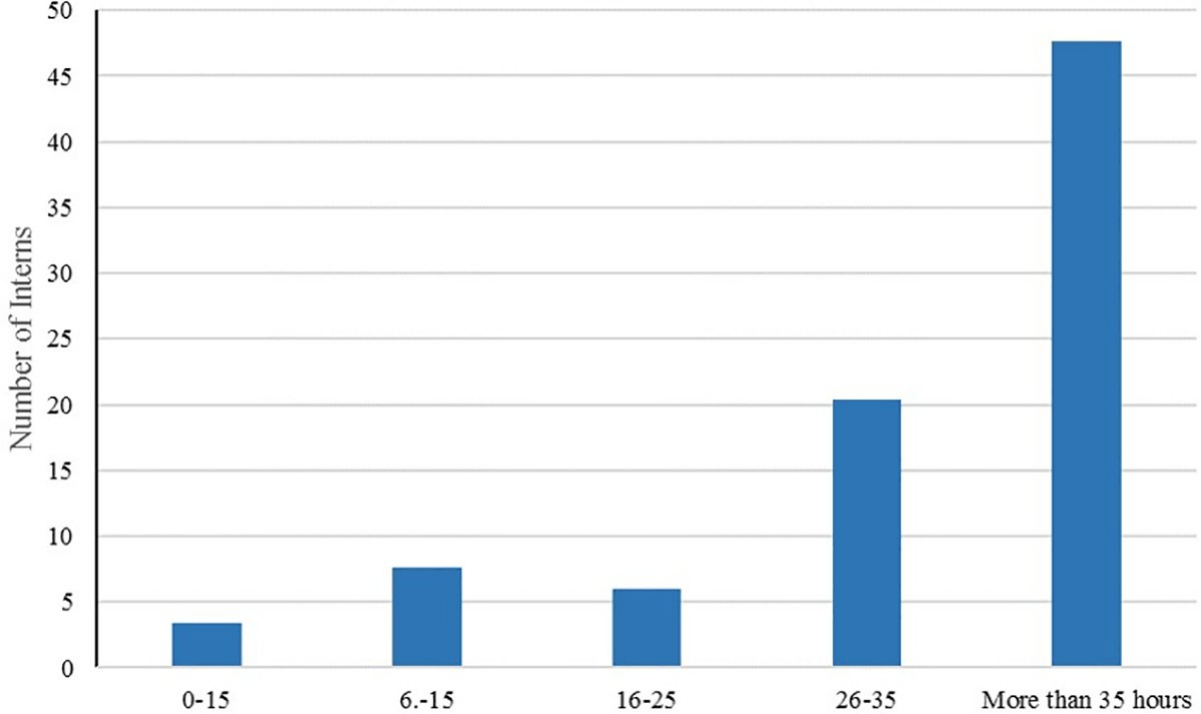
Bar chart showing the length of the international experience in hours.

Fig 5 indicates that 48 (56%) of interns dedicate more than 35 hours per week to international experience while the minority of respondents (4%) worked 0–5 hours weekly. About 9% of interns who completed the surveys worked an average of 6–15 hours a week. The findings further suggest that 7% and 24% of respondents worked an average of 16–25 and 26–35 hours weekly, respectively. Furthermore, results indicate that about 37% of respondents were a recipient of Humphreys Travel Grant (Humphreys Travel Grant provides funding to OSU students for international experiences of eight weeks or longer) while the rest did not receive these grants (63%).

**Fig 6:**
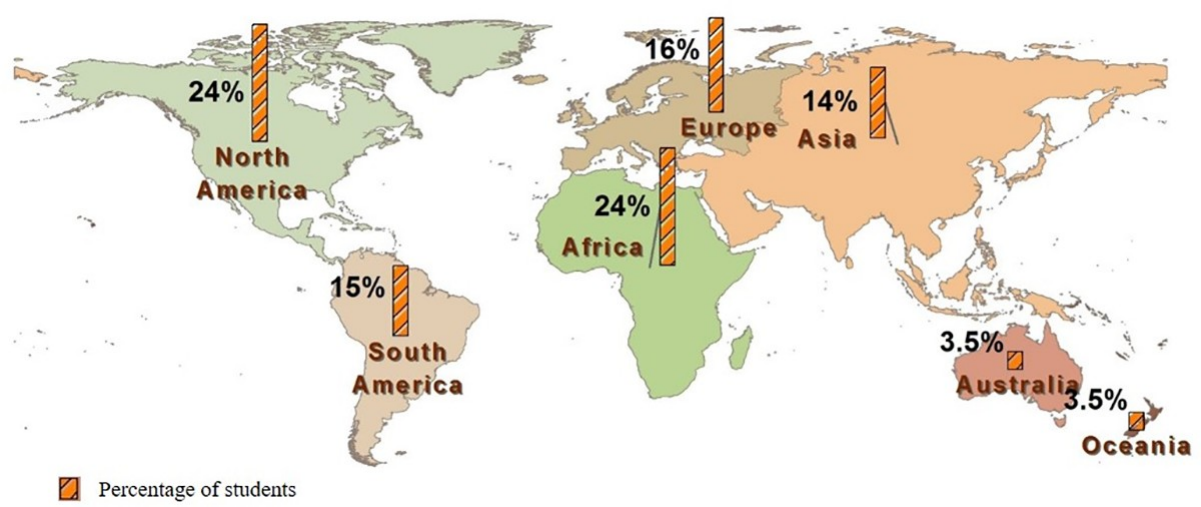
Location of host organizations by continents, percentage of total students in each continent.

**Fig 7:**
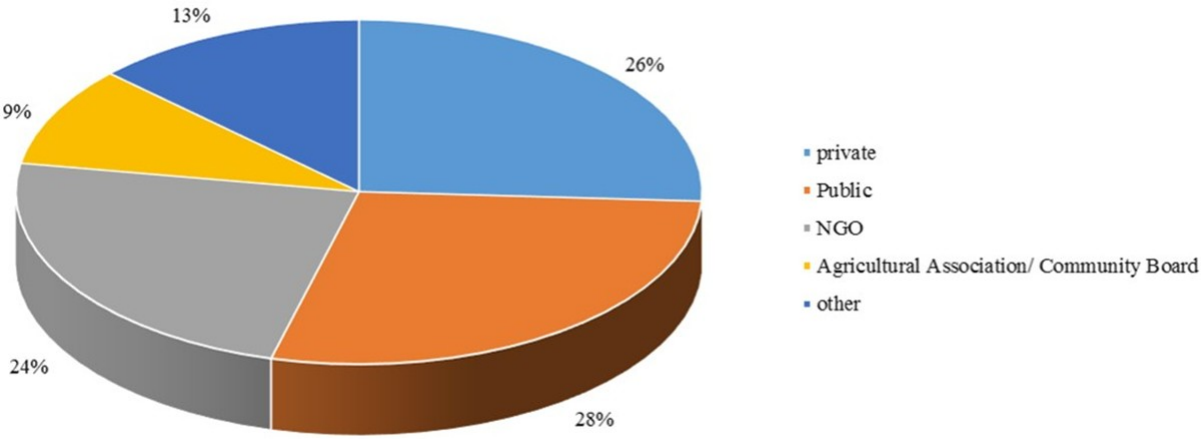
Type of host organizations.

Fig 6 indicates the continents and countries of the interns’ international experiences. As shown in Fig 5, Africa (24%), Europe (16%), North America (20%), and South America (14%) respectively, had the highest of share in hosting the MIAP interns, while Asia and Australia/Oceania had the lowest share (8%). In addition, the results show that two countries Uganda (15%) and Mexico (7%) were selected by more interns than other countries for their international experience. From Fig 6, it can be seen that 24% of student respondents have completed their internships in the North American continent (US, Canada, and Mexico). Of these, 5% of the total number of student respondents had completed their internships in the United States. These 5% are the percentages of respondents that are international and completed their internships in the US. So, this is another reason why the sample could be considered as a representative of the MIAP population. Fig 7 shows the type of host organizations. As shown in Fig 6, about half of respondents take their internship in public and NGO organizations.

To the best of our knowledge, almost all of the internships outside the US were unpaid. Globally, most of the host country government immigration rules prohibit payment to students with temporary tourist visas. Only a very small percentage of the MIAP total student interns (sixteen students) were paid a small stipend through a contract between the Noble Research Institute and OSU for the Uganda internships. Only 13 of the survey respondents indicated Uganda as their country of destination, which accounts for only 15% percentage of total respondents. We at the MIAP administrative office have not been informed of any other internships that any of our students completed that they were received any monetary payments. Although some students received in-kind payment. Examples are subsidized housing and meals that are difficult to attach a value to.

### The intern’s perceived impacts

**Fig 8:**
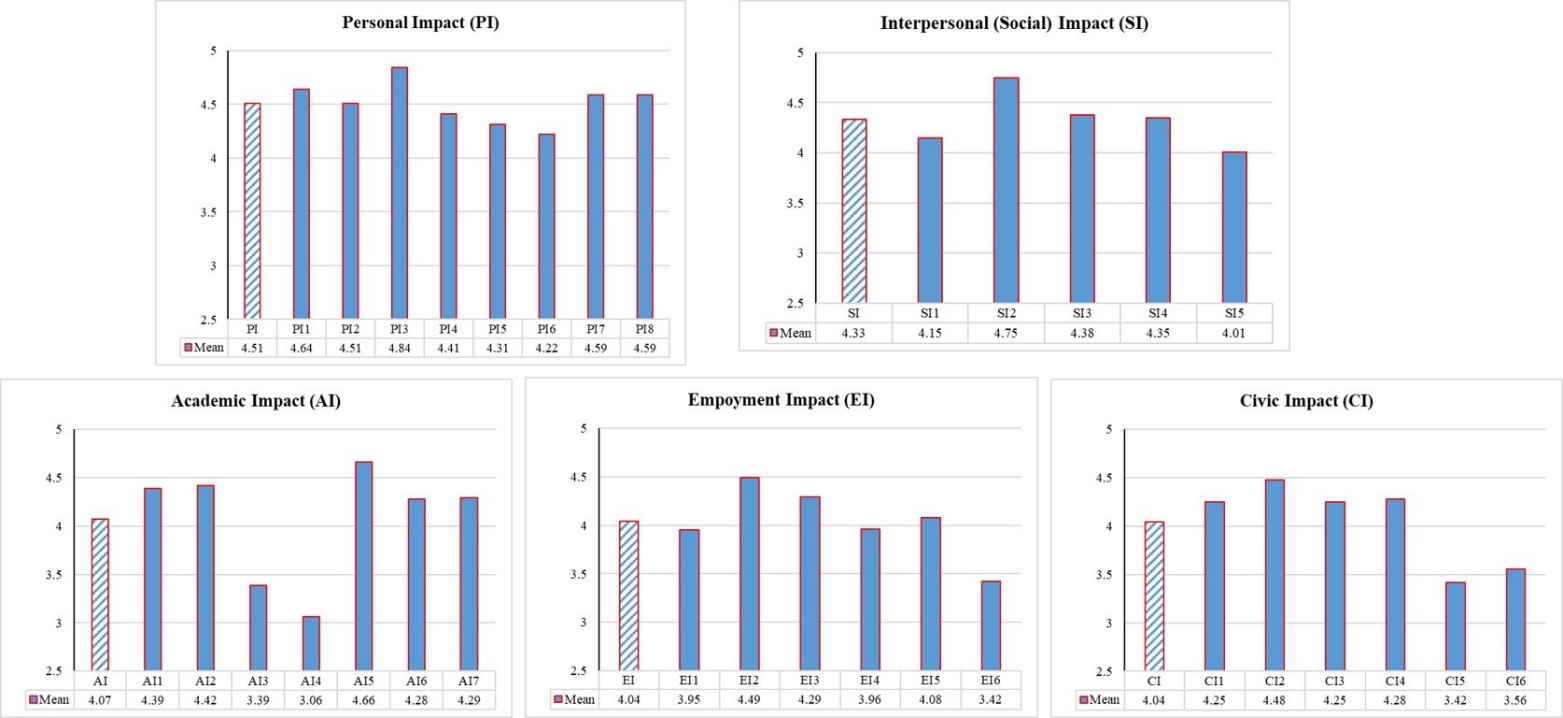
Summary statistics of the internship impacts.

Fig 8 shows that the mean for the thirty-two internship benefits (item) and the five international experiences impact. The results of internship benefits indicate that interns believe that international experience had enhanced each of the abilities. Among items, PI3 “I am open to new experiences” is reported as the highest achieved benefit of international experience (mean = 4.84), while AI4 “I desired to continue my education for another master or a Ph.D. degree” is reported as the lowest achieved benefit of international experience (mean = 3.06).

In addition, according to the results of Fig 6, interns believe that international experience has a critical role in improving the PI, SI, AI, EI, and CI. Interns reported that the highest and lowest international experience benefit related to PI (mean = 4.51), AI and CI (mean = 4.04), respectively.

### Evaluation of PLS-SEM

The evaluation of PLS-SEM results consists of two main steps. Step 1 investigates the measurement of models; if the results of measurement model are reliable, we are able to move on to the next step which involves assessing the structural model [88, 89]. In the following, the results of these two steps are presented.

### Measurement model results

In PLS-SEM, the measurement model was assessed by three characteristics: (1) investigating item-reliability analysis; (2) coverage validity; (3) discriminant validity.

The item- reliability depicts the correlation of the items with related latent variables [90]. To assess the item-reliability, standardised loadings were evaluated that should be above the threshold of 0.7 [70]. Hence, items have loading below the threshold were omitted. The results of Table 2 indicates that all remaining items loading are greater than the recommended value.

**Table 2:** Measurement model: Reliability and convergent validity.

Constructs	Items	Factor Loading	rho_A	Composite reliability (Dillon-Goldstein’s ρ)	Cronbach’s Alpha	AVE
**Academic impact model (sample size 85)**						
PI	PI1	0.78	0.92	0.93	0.92	0.67
	PI2	0.85				
	PI4	0.86				
	PI5	0.85				
	PI6	0.83				
	PI7	0.80				
	PI8	0.76				
SI	SI2	0.78	0.80	0.87	0.80	0.62
	SI3	0.81				
	SI4	0.85				
	SI5	0.71				
AI	AI1	0.85	0.84	0.89	0.83	0.67
	AI2	0.85				
	AI6	0.71				
	AI7	0.87				
Week	WE	1	1	1		
Hours	HO	1	1	1		
Humphreys	HU	1	1	1		
Supervisor	SU	1	1	1		
**Employment impact model (sample size 85)**						
PI	PI1	0.78	0.93	0.93	0.92	0.67
	PI2	0.86				
	PI4	0.86				
	PI5	0.85				
	PI6	0.82				
	PI7	0.80				
	PI8	0.76				
SI	SI2	0.763	0.80	0.87	0.80	0.62
	SI3	0.80				
	SI4	0.84				
	SI5	0.73				
EI	EI1	0.79	0.91	0.93	0.91	0.73
	EI2	0.83				
	EI3	0.90				
	EI4	0.86				
	EI5	0.88				
Week	WE	1	1	1		
Hours	HO	1	1	1		
Humphreys	HU	1	1	1		
Supervisor	SU	1	1	1		
**Civic impact model (sample size 85)**						
PI	PI1	0.78	0.92	0.93	0.92	0.67
	PI2	0.86				
	PI4	0.85				
	PI5	0.85				
	PI6	0.83				
	PI7	0.79				
	PI8	0.75				
SI	SI2	0.86	0.83	0.90	0.83	0.74
	SI3	0.87				
	SI4	0.85				
CI	CI1	0.89	0.89	0.92	0.89	0.75
	CI2	0.86				
	CI3	0.84				
	CI4	0.87				
Week	WE	1	1	1		
Hours	HO	1	1	1		
Humphreys	HU	1	1	1		
Supervisor	SU	1	1	1		
PEM	PE	1	1	1		

Source: Research results.

Coverage validity is estimated to ensure that the items of a latent variable which are theoretically related, are related in real condition. The coverage validity of the measured constructs is assessed by three tests: (1) Cronbach’s alpha, (2) composite reliability, and (3) average variance extracted (AVE) (4) rho_A. In an adequate model, composite reliability, Cronbach’s alpha [91], and rho_A [92] should be greater than 0.7. According to the results of Table 2, the scores of composite reliability and Cronbach’s alpha are above the recommended threshold. Besides, the results indicate that AVE for each latent variables exceeds the recommended threshold of 0.5 [93].

Discriminant validity is estimated to investigate the degree of divergence among latent variables. Discriminant validity is evaluated based on two criteria: (1) square root of AVE score, and (2) cross-loading [94]. In an adequate model, the square root of AVE of each construct should be greater than the correlation of two constructs. Based on the results of Table 3, the square root of AVE of each construct (on-diagonal cells) is higher the correlation between this construct and other constructs (off-diagonal cells). Based on the theory, loadings of each indicator on its construct should be greater that cross-loadings on other constructs [95]. Table 4 shows the results of cross-loadings for academic, employment, and civic impact models. Based on the findings of this table, discriminant validity of the latent variables was approved.

**Table 3:** Discriminant validity.

Constructs	PI	SI	AC	EM	CI	WE	HU	HO	SU	PE
**Academic impact model (sample size 85)**										
PI	**0.82**									
SI	0.63	**0.79**								
AI	0.38	0.33	**0.82**							
WE	0.15	0.13	0.05			**1**				
HU	0.02	0.03	0.01			0.04	**1**			
HO	0.06	0.04	0.04			0.16	0.02	**1**		
SU	0.04	0.01	0.02			0.01	0.00	0.05	1	
**Employment impact model (sample size 85)**										
PI	**0.82**									
SI	0.63	**0.79**								
EI	0.29	0.21		**0.85**						
WE	0.15	0.13		0.13		**1**				
HU	0.02	0.06		0.01		0.04	**1**			
HO	0.06	0.04		0.10		0.16	0.02	**1**		
SU	0.08	0.01		0.02		0.02	0.00	0.05	**1**	
**Civic impact model (sample size 85)**										
PI	**0.82**									
SI	0.51	**0.86**								
CI	0.56	0.31			**0.87**					
WE	0.15	0.09			0.06	**1**				
HU	0.02	0.06			0.05	0.04	**1**			
HO	0.06	0.03			0.04	0.16	0.02	**1**		
SU	0.04	0.008			0.04	0.01	0.00	0.05	**1**	
PE	0.02	0.03			0.05	0.01	0.05	0.00	0.02	**1**

Source: Research results.

**Table 4:** Cross loading.

**Constructs**	**PI**	**SI**	**AI**	**WE**	**HU**	**HO**	**SU**
**Academic impact model (sample size 85)**							
PI1	**0.78**	0.56	0.52	0.26	0.15	0.28	0.24
PI2	**0.85**	0.67	0.53	0.33	0.16	0.22	0.23
PI4	**0.86**	0.65	0.63	0.31	0.09	0.30	0.16
PI5	**0.85**	0.69	0.44	0.28	0.11	0.11	0.06
PI6	**0.85**	0.68	0.45	0.35	0.12	0.13	0.12
PI7	**0.79**	0.63	0.44	0.36	0.07	0.18	0.14
PI8	**0.76**	0.67	0.47	0.31	0.13	0.18	0.11
SI2	0.58	**0.78**	0.41	0.30	0.29	0.13	0.02
SI3	0.56	**0.81**	0.31	0.23	0.27	0.19	0.09
SI4	0.68	**0.85**	0.52	0.26	0.08	0.15	0.13
SI5	0.63	**0.71**	0.52	0.32	0.18	0.18	0.02
AI1	0.51	0.43	**0.85**	0.27	0.02	0.28	0.16
AI2	0.53	0.52	**0.85**	0.26	0.06	0.22	0.01
AI6	0.54	0.42	**0.71**	0.11	0.10	0.00	0.18
AI7	0.43	0.49	**0.87**	0.11	0.10	0.12	0.10
Week	0.38	0.36	0.23	**1.00**	0.19	0.41	0.12
Humphreys	0.14	0.25	0.08	0.19	**1.00**	0.15	0.00
Hours	0.25	0.21	0.19	0.41	0.15	**1.00**	0.23
Supervisor	0.19	0.08	0.14	0.12	0.00	0.23	**1.00**
**Constructs**	**PI**	**SI**	**EI**	**WE**	**HU**	**HO**	**SU**
**Employment impact model (sample size 85)**							
PI1	**0.78**	0.57	0.44	0.26	0.15	0.28	0.24
PI2	**0.86**	0.67	0.49	0.33	0.16	0.22	0.23
PI4	**0.86**	0.66	0.60	0.31	0.09	0.30	0.16
PI5	**0.85**	0.69	0.35	0.28	0.11	0.11	0.06
PI6	**0.82**	0.69	0.33	0.35	0.12	0.13	0.12
PI7	**0.80**	0.62	0.40	0.36	0.07	0.18	0.14
PI8	**0.76**	0.67	0.40	0.31	0.13	0.18	0.11
SI2	0.58	**0.76**	0.24	0.30	0.29	0.13	0.02
SI3	0.56	**0.80**	0.24	0.23	0.27	0.19	0.09
SI4	0.69	**0.84**	0.41	0.26	0.08	0.15	0.13
SI5	0.63	**0.73**	0.48	0.32	0.18	0.18	0.02
EI1	0.37	0.39	**0.79**	0.31	0.07	0.22	0.04
EI2	0.44	0.31	**0.83**	0.24	-0.06	0.38	0.18
EI3	0.50	0.41	**0.91**	0.26	0.08	0.25	0.18
EI4	0.48	0.41	**0.86**	0.33	0.10	0.22	0.12
EI5	0.50	0.43	**0.88**	0.38	0.05	0.31	0.11
Week	0.38	0.36	0.36	**1.00**	0.19	0.41	0.12
Humphreys	0.15	0.25	0.05	0.19	**1.00**	0.15	0.00
Hours	0.26	0.21	0.32	0.41	0.15	**1.00**	0.23
Supervisor	0.19	0.08	0.15	0.12	0.00	0.23	**1.00**
**Constructs**	**PI**	**SI**	**CI**	**WE**	**HU**	**HO**	**SU**	**PE**
**Civic impact model (sample size 85)**								
PI1	**0.78**	0.48	0.69	0.26	0.15	0.28	0.24	-0.08
PI2	**0.86**	0.60	0.66	0.33	0.16	0.22	0.23	-0.09
PI4	**0.86**	0.54	0.70	0.31	0.09	0.30	0.16	-0.04
PI5	**0.86**	0.66	0.61	0.28	0.11	0.11	0.06	-0.12
PI6	**0.83**	0.61	0.62	0.35	0.12	0.13	0.12	-0.21
PI7	**0.79**	0.61	0.49	0.36	0.07	0.18	0.14	-0.15
PI8	**0.75**	0.62	0.49	0.31	0.13	0.18	0.11	-0.06
SI2	0.58	**0.86**	0.52	0.30	0.29	0.13	0.02	-0.23
SI3	0.56	**0.87**	0.37	0.23	0.27	0.19	0.09	-0.10
SI4	0.69	**0.85**	0.53	0.26	0.08	0.15	0.13	-0.12
CI1	0.68	0.46	**0.89**	0.14	0.18	0.11	0.18	-0.16
CI2	0.62	0.51	**0.86**	0.31	0.21	0.27	0.24	-0.27
CI3	0.63	0.41	**0.84**	0.29	0.20	0.24	0.18	-0.19
CI4	0.66	0.54	**0.87**	0.13	0.17	0.05	0.14	-0.13
Week	0.38	0.31	0.25	**1.00**	0.19	0.41	0.12	0.12
Humphreys	0.15	0.25	0.22	0.19	**1.00**	0.15	0.00	-0.23
Hours	0.25	0.18	0.19	0.41	0.15	**1.00**	0.23	-0.01
Supervisor	0.19	0.09	0.21	0.12	0.00	0.23	**1.00**	-0.13
PEM	-0.13	-0.18	-0.22	0.12	-0.23	-0.01	-0.13	**1.00**

According to the findings of this section, we are able to conclude that the measurement models were reliable.

### Analysis of structural model

Table 5 indicates the findings of the structural model for academic, employment, and civic impact models. According to the results of academic impact model, PI has a positive and significant effect on AI (β = 0.44; P < 0.01). WE has significant effect on PI (β = 0.33; P < 0.01). SI has a positive and significant impact on AI (β = 0.23; P < 0.1). WE has significant effect on SI (β = 0.30; P < 0.01). HU has a positive and significant impact on SI (β = 0.19; P < 0.05). While, direct effect of SU on PI, HO on PI, HO on SI, HO on AI are not significant. In addition, the indirect effects of WE on AI (WE → SI → AI), HU on AI (HU → SI → AI), SU on AI (SU → SI → AI), and HO on AI (HO → SI → AI and HO → SI → AI) are not significant. While, indirect effects of WE on AI (WE → PI → AI) is positive and significant (β = 0.14; P < 0.05), that clearly indicates the full mediation effect, this result support H1.

**Table 5:** Structural model results.

	Direct effect	Indirect effect
**Causal Path**	β	β
**Academic impact model (sample size 85)**		
PI → AI	0.44***	
WE → PI	0.33***	
WE → PI → AI		0.14**
SI → AI	0.23*	
WE → SI	0.30***	
WE → AI	-0.04	
WE → SI → AI		0.07
HU → SI	0.19**	
HU → SI → AI		0.04
SU→ PI	0.13	
SU → SI → AI		0.05
HO → PI	0.09	
HO → PI → AI		0.03
HO → SI	0.06	
HO → AI	0.05	
HO → SI → AI		0.01
**Employment impact model (sample size 85)**		
PI → EI	0.41**	
WE → PI	0.33***	
WE → PI → EI		0.14**
SI → EI	0.06	
WE → SI	0.30***	
WE → EI	0.11	
WE → SI → EI		0.02
HU → SI	0.19*	
HU → SI → EI		0.01
HO → EI	0.16**	
HO → PI → EI		0.03
HO → SI	0.09	
HO → PI	0.06	
HO → SI → EI		0.003
SU→ PI	0.13	
SU → SI → EI		0.05
**Civil impact model (sample size 85)**		
PI → CI	0.72****	
WE → PI	0.33***	
WE → PI → CI		0.24*
SI → CI	0.04	
WE → SI	0.25**	
WE → CI	-0.05	
WE → SI → CI		0.004
HU → CI	0.10	
HU → SI	0.19**	
HO → CI	-0.005	
HO → PI → CI		0.06
HO → PI	0.09	
HO → SI	0.05	
HO → SI → CI		0.001
HU → SI → CI		0.003
SU→ PI	0.13	
SU → PI → CI		0.09
SU→ CI	0.07	
PE→ CI	-0.08	

*, ** and *** respectively, significance at the 10%, 5% and 1% levels.

Source: research results.

The findings of the employment impact model show that PI has significant effect on EI (β = 0.41; P < 0.05). Also, WE has significant effect on PI (β = 0.33; P < 0.01). WE has a positive and significant impact on SI (β = 0.30; P < 0.01). HU has a positive and significant effect on SI (β = 0.19; P < 0.10). HO has a positive and significant effect on EI (β = 0.16; P < 0.05). While, direct effect of SI on EI, WE on EI, HO on SI, HO on PI, SU on PI are not significant. In addition, the indirect effects of WE on EI (WE → SI → EI), HU on EI (HU → SI → EI), SU on EI (SU → SI → EI), and HO on EI (HO → SI → EI and HO → SI → EI) are not significant. While, indirect effects of WE on EI (WE → PI → EI) is positive and significant (β = 0.14; P < 0.05), that clearly indicates the full mediation effect, this result support H2.

The results of the civic impact model indicate that PI has a positive and significant effect on CI (β = 0.72; P < 0.01). WE has significant effect on PI (β = 0.33; P < 0.01). WE has a positive and statistically significant impact on SI (β = 0. 25; P < 0.05). HU has a positive and significant effect on SI (β = 0. 19; P < 0.05). While, direct effect of SI on CI, WE on CI, HO on SI, HO on PI, HO on CI, SU on PI, SU on CI, HU on CI, and PE on CI are not significant. Furthermore, the indirect effects of WE on CI (WE → SI → CI), HO on CI (HO → PI → CI and HO → SI → CI), and HU on CI (HU → PI → CI) are not significant. While, indirect effects of WE on CI (WE → PI → CI) is positive and significant (β = 0.24; P < 0.1), that clearly indicates the full mediation effect, this result support H3.

Although overall goodness-of-fit indices are not generated by the PLS-SEM [96], Tenenhaus, Vinzi (98) has presented a Goodness of Fit (GOF) index (0<GOF<1) to evaluate the model fit. For calculating the GOF index, the following equation is employed (*GOF* = √*AVE*×*R*^2^), where AVE is the geometric mean of the average communality and *R*^2^ is the average of *R*^2^.

The threshold values for evaluating the results of GOF are defined as small (0.10), medium (0.25), and large (0.36) [97]. Based on the results of Table 6, GOF index values for academic impact model (0.42), employment impact model (0.41), and civic impact model (0.48) are greater of (0.36) that shows these models have a very good model fit.

**Table 6:** Calculation of Goodness of Fit (GOF) index.

Constructs	Value
**Academic impact model**	0.73
Average communality	0.25
Average R-squared	0.42
GOF	
**Employment impact model**	
Average communality	0.74
Average R-squared	0.23
GOF	0.41
**Civic impact model**	
Average communality	0.78
Average R-squared	0.30
GOF	0.48

*, ** and *** respectively, significance at the 10%, 5% and 1% levels.

Source: research results.

## Discussion and conclusions

The objective of this study is to evaluate the MIAP students’ international experiences and assess what factors were associated with international experience outcomes. Primary data are collected from an online survey distributed to the Master of International Agriculture Program (MIAP) graduates and current students who had completed an international experience/internship while a student in MIAP. The survey has three main parts: demographics, internship provider information, and internship perceived impacts on MIAP students. The last part (internship impacts on students) is divided into five main category of questions, each containing sub-category questions. The response rate to the survey was high (66%), which might point to the connection of students to their internships and their degree program (MIAP).

Results show that half of the students chose internships in two fields: agricultural extension and agribusiness development. This might be indicative of students’ interest in gaining more experience in these fields to prepare themselves for related careers for after their graduation. It is important to note that students choose their internships and the areas of internship work from a wide range of internships that MIAP offers or they themselves choose from their search results.

The results regarding the internship length and hours per week worked might indicate the students’ commitment to international work as well as their desire to learn from a hands-on experience. The length of stay results might also be a consequence of the funding, which 37% of students had received. Note that Humphreys funding is for international experiences of eight weeks or longer and that might have influenced the students’ choice regarding the length of their internship. Last, but not least, is the long-term impact of international experience/internship as perceived by the MIAP graduates. Survey results show that students perceive their international experience as having a significantly positive impact on them as measured by the personal impact of 4.51 from a maximum of five, the interpersonal impact of 4.33, the academic impact of 4.07, employment (job specific) impact of 4.04, and civic impact of 4.04 (Fig 6). It is important to note that the impact factor of more than 3 indicates that the respondents’ perception of the impact of the internship is positive. The closer the impact factor to five, the higher the perceived impact.

Research hypothesis 1 was that the personal and interpersonal (social) impacts have a mediation effect on the relationship between student intern/internship characteristics and academic outcomes. The results of this study support the hypothesis, finding that the indirect effects of length of internship on academic outcomes through personal impacts (length of internship → personal impacts → academic outcomes) is positive and significant, which clearly indicates the full mediation effect. Research hypothesis 2 was that the personal and interpersonal (social) impacts have a mediation effect on the relationship between student intern/internship characteristics and employment outcomes. The statistical findings also support this hypothesis, finding that the indirect effects of length of internship on employment outcomes (length of internship → personal impacts → employment outcomes) is positive and statistically significant, that clearly indicates the full mediation effect. Research hypothesis 3 was that the personal and interpersonal (social) impacts have a mediation effect on the relationship between student intern/internship characteristics and civic outcomes. Again, the findings support this hypothesis, showing the indirect effects of length of internship on civic outcomes (length of internship → personal impacts → civic outcomes) is positive and statistically significant, which clearly is an indicative of the full mediation effect. More specifically, respondents believe that their preparedness for their professional positions in terms of their applied, hands-on-knowledge as well as technical skills was significantly improved because of their international experience. Moreover, the graduates feel that their international experience played a significant role in the graduates developing a realistic idea about their work load at their professional position and helped them narrow down future possible career choices.

The findings of this study indicate that internship characteristics have a stronger effect compared with student intern characteristics on international experience impacts, which is consistent with the results of Jackel (44). These results indicate that the MIAP planners and decision makers could improve the students’ effectiveness through setting the internship characteristics such as length of internship, Humphreys travel grants, and the number of hours worked weekly.

The findings related to academic and employment impact model showed that Humphreys travel grants has a positive and significant direct effect on social impacts. This result is consistent with the findings of Weidman and Stein [66], who argue that when the level of financial support is high, students tend to enhance their commitment and responsibility to the members of their research team. Attfield and Couture [67] show that financial concerns could be as one of the most important reasons that prevent students from having the opportunity to better connect with the communities that they serve during their internship.

The research also confirms length of internship is significantly associated with social impacts and personal impacts in the academic, employment, and civic impacts models, which are consistent with the similar findings in the literature [61, 63, 98]. Petrillose and Montgomery [62] point out that an increase in the internship length leads to students acquiring more benefits from their internship. Especially, students who participate in international experiences need to have enough time to overcome the internship challenges such as cultural transition, language barrier, relationships, etc. Hence, length of internship is one of the most important and effective factors in improving personal and social outcomes. The number of hours worked weekly at internship is found significant and positively related to employment outcomes. This is also consistent with the literature [15].

The findings of academic, employment, civic impacts models show that interns who experienced more personal impacts, demonstrated a higher level of academic, employment and civic outcomes, respectively as compared to those who did not. The academic impact model shows that students who experienced more personal and social impacts, showed more academic impacts.

Almost 79% of respondents indicated that they would have participated in an internship, even if it were not required. This finding might indicate that interns have an accurate awareness of the importance of the important role that an international experience has in improving their personal, social, academic, employment, and civic status. Hence, we suggest that the MIAP planners and decision-makers provide appropriate opportunities for interns to increase their internship length, without financial and academic concerns. This goal could be achieved by increasing the financial support and changing the curriculum requiring longer international experiences (eight weeks or longer, rather than four weeks or longer).

This study contributes to the existing literature in its use of a statistical technique (Structural Equation Modeling, SEM) to analyze the influence of an educational component, international experience part of a master degree, on student learning as well as impacts on social, civic, employment, and personal impacts. To the best of the authors’ knowledge and from a thorough review of the existing literature, this is the first study that has used the SEM technique to analyze the impact of internships on students. This method’s superiority is in its ability to handle complex direct and indirect statistical relationships, as seen in this study between internship characteristics and effects on students.

Since the international experience program has several stakeholders who pursue their own organizational goals, future research should also receive feedback from MIAP administrators, employers, host organizations, and Humphreys Chairs that could indeed provide useful information to identify the strengths and weaknesses of this program.

It can be concluded that internships that come with funding and are of longer term allow students to have the most impact on the communities that they serve in through their internships as well as on the students themselves. Therefore, it would be wise for international program officers and internship organizers at academic institutions to focus their efforts on fund raising and organizing longer-term internships in agricultural development. In order to accommodate students and attract them to longer term internships, administrators might consider devoting a semester to international experiences when designing curriculum. This might require offering online courses that students can take during their international experiences so that they are not delayed in completing their degree programs.

### Limitations and implications for future research

One of the major shortcomings of this study involves data limitations. Students’ contact information changes overtime and many do not update their information with their college, which limits the response rate. To increase the response rate, we recommend that colleges/programs send any surveys to their students towards the end of the student internships or after they return, but before they would have graduated. In addition, we recommend utilizing the email lists of alumni associations in colleges and universities for research conduct to help with having access to current alumni contact information. Also, we encourage forming or strengthening alumni associations at the departmental levels for current contact with the students. In this study, we could have significantly increased the number of observations for the statistical analysis, have we had access to current contact information through an active MIAP alumni association. One of the primary limitations of the study is that the data is self-reported and as humans we do not always know ourselves as well as we think we do. Another is that those students who elected to complete the survey may be those for whom the internship experience was most meaningful. In this study, we did not include data from the students who completed their internships domestically due to health, financial, employment status, or other limitations that prevented them from going abroad. A worthwhile expansion of this study would be to include an analysis related to the domestic internships and compare the impact results with those from international internships. Another interesting comparison criterion would be by the destination continent or world geographic region where the internships are located in. Because of data limitation as pointed out in the previous paragraph, we were not able to do the comparison of impacts using this criterion (domestic versus international).

Because the type of the internship work that the student participant engages in is varied, it would be interesting to compare the impact results by the internship type in a future study. More specifically, it would be interesting to compare impacts from internships that involve theoretical or scientific/testing laboratory type work with those that are of a more practical, hands-on nature. Also comparing impacts by type of the communities that the students serve in through their internships might be a worthy future study. For example, to examine whether students working with youth, women, and underserved communities in rural areas perceive as having a larger impact than those serving in urban settings working with higher income population. Example of the former being students helping with setting up a women’s saving circle, a small-farmer association, or to improve educational and technical skills of women and youth versus those conducting internships in a botanical garden, a Zoo, with commercial farms, or working with an NGO to save the whales or sea turtles.

One of the limitations of this study is that it focuses solely on the structures (duration, location, etc.) of the internship and not necessarily on the educational curriculum, facilitation, sense-making, or anything else that might be going on inside the curriculum that the MIAP students undertake during their internships. Therefore, it is recommended for future studies to include the impact of content and type of internships.

One of the real risks of international service-learning is that it can sometimes be much more beneficial to the students than to the organizations and communities that are supposed to be benefiting from the service. Therefore, it would be valuable to internship designers and academic program administrators if future studies included a a survey to receive feedback from the host organizations/host communities related to the hosts’ perception of student impact.

## Supporting information

S1 File Do-file containing Stata commands (executes in Stata 15.1 with catplot installed). (DO)

S2 File Do-file containing PLS-SEM program code (executes in Stata 15.1 with catplot installed). (DO)

S3 File (DO)

S1 Appendix Person correlation for personal, internship characteristics, and internship impacts. (DOCX)

## Definition of abbreviations

AI: Academic impacts
AVE: Average variance extracted
CEM: Employment status in the current situation
CI: Civic impacts
EI: Employments impacts
GOF: Goodness of fit
HO: The number of hours worked weekly at internship
HU: Humphreys travel grants
INT: Interest in international experience
MIAP: Master of international agricultural degree program
PEM: Employment status during the internship
PI: Personal impacts
PLS-SEM: Partial least squares structural equation modeling
SI: Interpersonal impacts
SUP: Access to supervisor during internship
TRA: Preparation and or training received before placement
WE: Length of internship
