# Supplementary material for: Quantifying impacts of internships in an international agriculture degree program
Source: PLoS One. 2020 Aug 17;15(8):e0237437. doi: 10.1371/journal.pone.0237437 (PMC7430748; doi:10.1371/journal.pone.0237437)
Supplement: S1 Appendix — (DOCX) [file pone.0237437.s004.docx]

| **Variables** | **PI** | **SI** | **AI** | **EI** | **CI** |
| --- | --- | --- | --- | --- | --- |
| INT | 0.007 | 0.004 | 0.04 | 0.02 | 0.09 |
| PMI | -0.04 | -0.14 | -0.12 | 0.08 | -0.19^*^ |
| CMI | -0.03 | -0.08 | -0.10 | -0.05 | -0.13 |
| HO | 0.25^**^ | 0.18^*^ | 0.19^*^ | 0.32^***^ | 0.19^*^ |
| WE | 0.38^***^ | 0.31^***^ | 0.23^**^ | 0.36^***^ | 0.24^**^ |
| HU | 0.15 | 0.25^**^ | 0.08 | 0.22 | 0.22^**^ |
| SUP | 0.19^*^ | 0.09 | 0.13 | 0.15 | 0.21^**^ |
| TRA | 0.05 | 0.11 | 0.13 | 0.17 | 0.06 |
| ^*^, ^**^ and ^***^ respectively, significance at the 10%, 5% and 1% levels. | | | | | |
